# Theoretical investigation of steric effects on the S1 potential energy surface of o-carborane-anthracene derivatives

**DOI:** 10.55730/1300-0527.3567

**Published:** 2023-05-22

**Authors:** Fahri ALKAN

**Affiliations:** Department of Nanotechnology Engineering, Faculty of Engineering, Abdullah Gül University, Kayseri, Turkey

**Keywords:** Carboranes, steric effects, excited-states, potential energy surface, TDDFT

## Abstract

TDDFT scan calculations were performed for s-carborane-anthracene derivatives (*o-*CB*-*X*-Ant* where X=-H, -CH_3_, -C_2_H_5_ and *tert*-butyl or -*t*Bu) in order to understand the interplay between the steric effects, S_1_ potential energy surface (PES) and photophysical properties. The results show that all systems exhibit three local minima on the S_1_ PES, which correspond to the emissive LE and TICT state, along with the nonemissive CT state respectively. In the case of the unsubstituted system (*o-*CB*-*H*-Ant*), and *-*CH_3_ and *-*C_2_H_5_ substituted cases, S_1_ PES is predicted to be quite flat for certain conformations indicating that it is possible for these systems to reach the nonemissive CT state without a large energy penalty. In comparison, conformational pathways for the nonemissive CT state are predicted to be energetically unfavorable for *o-*CB*-t*Bu*-Ant* as a result of both steric and electronic effects. These results provide a mechanism for the enhanced emission of σ-CB-fluorophore molecules with bulky ligands.

## 1. Introduction

Icosahedral carborane clusters (C_2_B_10_H_12_) show unique electronic and structural properties due to possessing three-center two electron bonds and s-aromaticity [1,2]. In recent years, significant progress has been made in C-H or B-H functionalization of carboranes, which enables carborane clusters to be employed as building blocks for nanoclusters, opto-electronic devices, and luminescent materials [3–6]. Among the possible isomers, σ-carborane (σ-CB) is the most widely-studied system due to its stability and relative ease of functionalization. In particular, σ-CB-fluorophore architecture has attracted interest due to its distinct photophysical properties such as aggregation-induced emission (AIE) and multiple photoluminescence [1,7–15].

As shown previously, σ-CB-fluorophores can exhibit dual emission in solution from locally-exited (LE) and twisted intramolecular charge transfer (TICT) states as a result of the conformational flexibility of σ-CB-fluorophore [16–20]. In the case of LE state, S_1_ →S_0_ (or S_0_→S_1_) transition is localized on the p-conjugated fluorophore, while TICT state exhibits the extension of p-conjugation through mixing of LUMO orbitals from σ-CB and the fluorophore moieties [16,17]. It is also shown that the orbital mixing becomes symmetrically largest with the perpendicular arrangement of σ-CB’s C-C bond (C_1_-C_2_) relative to the plane of fluorophore’s p system, and energetically favorable with the elongation of the same C_1_-C_2_ bond [21,22].

In solution state, σ-CB-fluorophore systems generally show poor emission quantum yields, [1,7,23,24] which is often associated with the nonradiative decay path resulting from the vibrational motion of C_1_-C_2_ bond [25,26]. More recently, it is shown that a nonemissive and energetically favorable charge-transfer (CT) state occurs on the S_1_ potential energy surface (PES) of σ-CB-fluorophore systems as a result of significant C_1_-C_2_ bond elongation, which can also be a major pathway for nonradiative decay [22,27]. In comparison, the emission is shown to be recovered in solid state systems (AIE), or via structural limitations on C_1_-C_2_ bond elongation [9,16,18,28–35]. It should also be noted that enhanced emission quantum yields have been obtained in solution for σ-CB-fluorophore molecules when bulky ligands are introduced to the adjacent carbon of σ-CB [12,17,26,36–39]. However, the mechanism for this enhanced emission and the effect of steric hindrance on the electronic structure and excited state properties of such systems have not been fully explored using theoretical tools.

In this contribution, a detailed investigation for the S_1_ PES of σ-CB-anthracene derivatives (*o-*CB*-*X*-Ant* where X=-H, -CH_3_, -C_2_H_5_ and *tert*-butyl or -*t*Bu) is presented to understand the steric effects on the photophysical properties of σ-CB-fluorophore molecules. The results show that while the nonemissive CT state is energetically favorable for *o-*CB-H-*Ant*, emissive TICT state becomes significantly more favorable with -*t*Bu substitution. It is also shown that energy barriers on S_1_ PES for the conformations exhibiting parallel orientation of C_1_-C_2_ bond and *Ant* are quite large with -*t*Bu substitution compared to the cases in X =-H, -CH_3_ and -C_2_H_5_. These findings provide a possible mechanism for the enhanced emission of σ-CB-fluorophore molecules with bulky ligands.

## 2. Materials and methods

**Figure 1:**
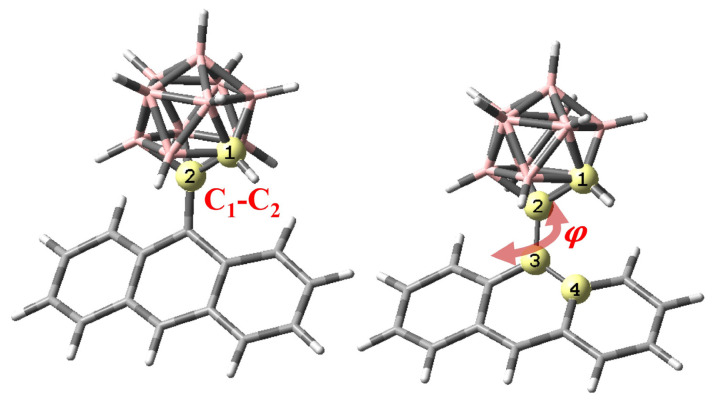
Illustration of C_1_-C_2_ bond length and the dihedral angle (ϕ) between C_1_-C_2_ bond and the plane of *Ant* moiety (C_1_-C_2_-C_3_-C_4_) for *o-*CB*-*X*-Ant*.

All DFT and TDDFT computations were performed with Gaussian09 program package [40] using M06-2X [41] functional and 6-31g(d) basis set. In previous work, M06-2X functional is shown to provide better agreement with experiment for geometries and excited state properties compared to other functionals [22,42]. In addition, benchmark calculations on *o-*CB-H-*Ant* system with 6-311g(d) basis set were performed. It is seen that exited state energies differ only by 0.06–0.03 eV for LE, TICT, and CT states, indicating that 6-31g(d) provide sufficient accuracy for these systems. For TDDFT scan calculations, excited-state geometry optimizations were performed with constraints on C_1_-C_2_ bond length, and the dihedral angle between C_1_-C_2_ bond and the plane of *Ant* moiety, which are illustrated in Figure 1. Excited-state density differences and L values, which quantify the overlap between electron and hole wavefunctions for an excited state, were calculated using Multiwfn [43] program.

Solvent effects were considered with THF as solvent within the polarizable continuum formalism. In previous work, [44] solvent effects are shown to be small but noticeable for photophysical properties of σ-CB-fluorophore systems. In that aspect, benchmark calculations were performed for *o-*CB-H-*Ant* with toluene and acetonitrile as solvents in addition to THF. It is seen that while solvent effects are somewhat smaller (~0.05 eV) for the LE state, TICT and CT states become considerably stable (~0.2 eV) with acetonitrile as solvent compared to the case with toluene due to charge transfer nature of the excited states.

## 3. Results and discussion

**Figure 2:**
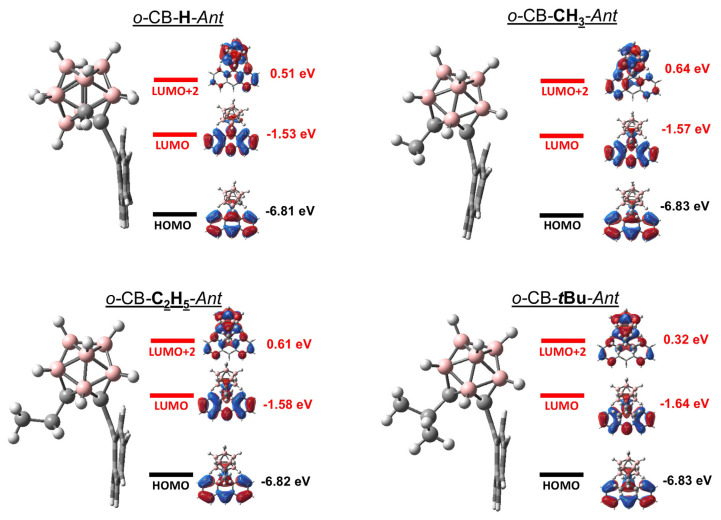
Ground state geometries and selected energy levels for σ-CB-anthracene derivatives.

Figure 2 shows the optimized ground state geometries, and the frontier energy levels which contribute significantly to the excited-state dynamics for *o-*CB-X-*Ant*. For ground state geometries, *o-*CB-H-*Ant* exhibits a −15° dihedral angle (ϕ), whereas the substituted systems (*o-*CB*-*CH_3_*-Ant*, *o-*CB*-*C_2_H_5_*-Ant* and *o-*CB*-t*Bu*-Ant*) exhibit tilted ϕ ranging between −86° and −88°. This result originates from the fact that steric effects are quite substantial even with the -CH_3_ substitution for small ϕ and unstretched C_1_-C_2_ bond. It is seen that both HOMO and LUMO levels are mainly localized on the *Ant* moiety, while LUMO+2 level exhibits significant contribution from *o-*CB in all cases. It should also be noted that the LUMO+2 level exhibits large antibonding character on the C_1_-C_2_ bond. As a result, this level is shown to undergo significant stabilization with increasing C_1_-C_2_ bond length and ordering of LUMO and LUMO+2 can be altered upon excitation induced geometry change [22,27].

In Table 1, the excited state energetics, oscillator strengths, and geometric parameters for vertical 0→1 transition (absorption), along with 1→0 transitions (emission) for LE, TICT, and CT states are tabulated. The geometries show only slight changes from the ground-state geometries for the 1→0 transitions of LE state. Regardless of this slight conformational change, both 0→1 transitions and 1→0 transitions in LE state originate from local π-π* transitions on the *Ant* moiety for all systems, while the contribution from *o-*CB is minimal. As a result, calculated oscillator strengths and energies (*f*) show quite similar values due to similar nature of the transition for these systems.

For the case of TICT state, it is previously shown that *o-*CB-H-*Ant* undergoes to a rotation with respect to *ϕ*, along with C_1_-C_2_ bond elongation compared to the LE or S_0_ states [16]. For the substituted systems, the conformations for TICT state also exhibit a tilted *ϕ* and partially elongated C_1_-C_2_ bonds. The elongation of C_1_-C_2_ bond is slightly less for *o-*CB*-*CH_3_*-Ant* and *o-*CB*-*C_2_H_5_*-Ant* (2.11 and 2.14 Å respectively), while the same bond length becomes substantially larger for *o-*CB*-t*Bu*-Ant* (2.35 Å). Furthermore, TICT state of *o-*CB*-t*Bu*-Ant* shows significant stabilization (0.31 eV) for the adiabatic energy (E_S1_) compared to LE state. In comparison, calculated E_S1_ is quite comparable for LE and TICT states of other molecules. In all cases, TICT state exhibits an increase for the contribution of *o-*CB moiety to electron wavefunction along with an increase for the oscillator strengths showing hybridized local and charge transfer (HLCT) character.

In our recent work, it is seen that significant elongation of C_1_-C_2_ bond and parallel orientation between two moieties (*ϕ* = 0°) result in a nonemissive CT state for *o-*CB-H-*Ant*.[22] Furthermore, this CT state is shown to be the global minimum for the S_1_ PES, which suggests that it can be an important pathway for fluorescence quenching. A similar dark CT state is also found for all substituted systems as shown in Table 1. In the case of *o-*CB*-*CH_3_*-Ant* and *o-*CB*-*C_2_H_5_*-Ant*, C_1_-C_2_ bond length becomes 2.59 Å for the dark CT state, while the same C_1_-C_2_ bond becomes even more elongated for *o-*CB*-t*Bu*-Ant* (2.68 Å), which most likely results from the steric hindrance of bulky -*t*Bu group. Another important point is that while the E_S1_ of the CT state is ~0.2 eV lower compared to the E_S1_ of the TICT state of *o-*CB-H-*Ant*, the same CT state is energetically less favorable (~0.2 eV) in the case of *o-*CB*-t*Bu*-Ant*. For *o-*CB*-*CH_3_*-Ant* and *o-*CB*-*C_2_H_5_*-Ant*, the energy differences between two states are less pronounced for these systems compared to the cases in *o-*CB*-t*Bu*-Ant* or *o-*CB-H-*Ant*.

**Figure 3:**
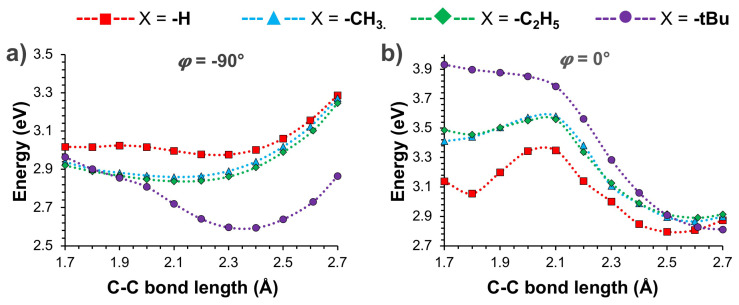
Calculated PESs for the S_1_ state of *o-*CB*-*X*-Ant* systems with respect to varying C_1_-C_2_ bond length, and fixed *j* at a) −90° and b) 0°.

To further understand the steric effects on the photophysical properties of *o-*CB*-*X*-Ant* systems, the PESs of the S_1_ state are scanned with respect to C_1_-C_2_ bond length and *ϕ*. In Figure 3a and 3b, the results are shown for conformations with varying C_1_-C_2_ bond length, and fixed *ϕ* (−90° and 0°) respectively. For the tilted geometries (*ϕ* = −90°), elongation of C_1_-C_2_ bond results in relatively flat PESs for the 1.7–2.4 Å range in the case of *o-*CB-H-*Ant* as shown in Figure 3a. Similar results are seen for the substituted systems when X = -CH_3_ and -C_2_H_5_. For the latter, calculated E_S1_ shows an increase with further elongation of C_1_-C_2_ bond beyond 2.4 Å. In comparison, the PES of *o-*CB*-t*Bu*-Ant* shows a clear minimum between 2.3–2.4 Å, which roughly corresponds to the TICT (*ϕ* = −86°) state shown in Table 1. In this case, elongation of C_1_-C_2_ bond results in a decrease for E_S1_ for the 1.7–2.4 Å range, which arises from the steric strain relaxation of bulky *-t*Bu group.

For parallel geometries (*ϕ* = 0°), lowest energy points for the calculated E_S1_ are found with conformations showing fully elongated C_1_-C_2_ bond lengths, which also correspond to the dark CT states of these systems as shown in Table 1. For *o-*CB-H-*Ant*, PES shows an energy barrier of ~0.4 eV, where the local maximum is located at 2.1 Å. This is also the case for *o-*CB*-*CH_3_*-Ant* and *o-*CB*-*C_2_H_5_*-Ant*, however, the calculated energy barriers are somewhat smaller (~ 0.1 eV) with respect to shorter C_1_-C_2_ bonds. It should be noted that the steric effects are more pronounced for parallel conformations (*ϕ* = 0°) due to the alignment of -X group with anthracene moiety, and these steric effects mostly dominate the excited-state energetics for relatively short C_1_-C_2_ bonds. In fact, the most drastic result is seen for X = -*t*Bu case, for which the PES shows a large destabilization of E_S1_ as shown in Figure 3b. For this system, PES does not exhibit an energy barrier since calculated E_S1_ becomes continuously more stable with elongation of the C_1_-C_2_ bond.

**Figure 4:**
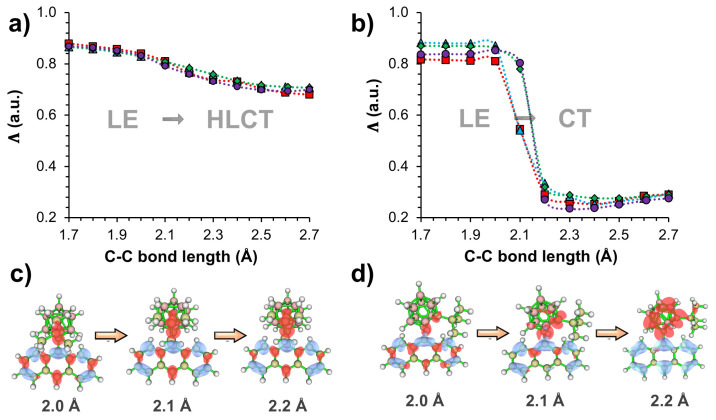
Calculated L values for a) tilted (*j* = −90°) and b) parallel conformations (*j* = 0°) with respect to C_1_-C_2_ bond lengths, and illustrations of excited-state density differences for c) tilted and d) parallel conformations at 2.0, 2.1, and 2.2 Å C_1_-C_2_ bond length.

In addition to the excited-state energetics, it is important to assess the nature of S_1_→S_0_ transitions on the PESs to fully understand the photophysical processes and the origin of energy barriers. In Figure 4a and 4b, calculated Λ values, which quantify the overlap degree (0 ≤ Λ ≤ 1) between electron and hole wave functions [45], are illustrated for the S_1_→S_0_ transitions of tilted (*ϕ* = −90°) and parallel conformations (*ϕ* = 0°) with respect to C_1_-C_2_ bond lengths. For both *ϕ*, S_1_→S_0_ transitions mainly originate from local π-π* transition on the anthracene moiety, and show LE character for shorter C_1_-C_2_ bond lengths with Λ larger than 0.8. In the case of tilted geometries, calculated Λ values become continuously smaller with increasing C_1_-C_2_ bond lengths, indicating more HLCT character for the S_1_→S_0_ transitions of all systems. This result originates from the larger *o-*CB character for the electron wave function with elongated C_1_-C_2_ bond lengths. This effect is also illustrated with excited-state density differences shown in Figure 4c for *o-*CB*-t*Bu*-Ant*. As the C_1_-C_2_ bond length elongates from 2.0 Å to 2.2 Å, electron density (shown as red) on *o-*CB gradually increases showing CT from *Ant* to *o-*CB upon excitation. Meanwhile, there is also considerable hole (shown as blue) and electron density on *Ant*, which indicates the presence of local π-π* character for the overall excited state nature.

For parallel conformations (*ϕ* = 0°), calculated Λ values (0.8–0.9) also exhibit LE character for the S_1_→S_0_ transitions when the C_1_-C_2_ bond length is within 1.7–2.1 Å. At 2.1 Å, however, the nature of S_1_→S_0_ transitions drastically transform to CT character as indicated from the sharp decrease of Λ values. This effect is also evident from excited-state density differences shown in Figure 4d. While electron and hole densities are mainly localized on *Ant* moiety when the C_1_-C_2_ bond length is 2.0 Å, they show a strong charge separation when C_1_-C_2_ bond length is 2.2 Å. It should also be noted that PESs (Figure 2b) show a significant decreasing trend for the calculated E_S1_ of all systems at 2.1 Å bond length, which is initiated with the LE→CT transformation of S_1_→S_0_ transitions.

**Figure 5:**
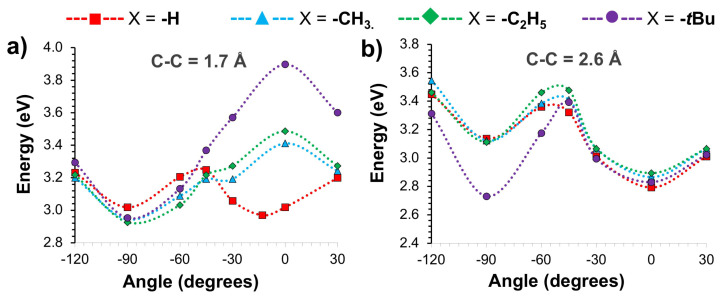
Calculated PESs for the S_1_ state of *o-*CB*-*X*-Ant* systems with respect to varying *j*, and fixed C_1_-C_2_ bond length at a) 1.7 Å and b) 2.6 Å.

Similar to the C_1_-C_2_ bond elongation, the PESs of the S_1_ states are also scanned with respect to *j* for selected bond lengths (1.7 or 2.6 Å). The results are illustrated in Figure 5a and 5b, respectively.

**Figure 6:**
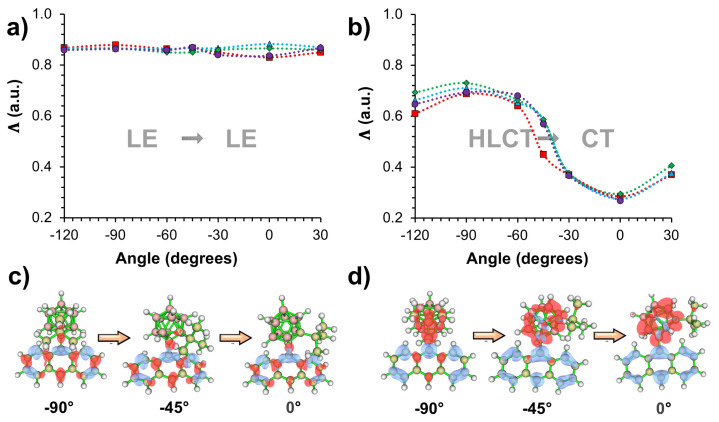
Calculated Λ values with varying *j* for conformations with a) unstretched (C_1_-C_2_ = 1.7 Å) and b) fully stretched (C_1_-C_2_ = 2.6 Å) C_1_-C_2_ bonds, and illustrations of excited-state density differences for c) unstretched and d) fully stretched conformations at −90°, −45°, and 0° dihedral angles.

For the unsubstituted system, calculated PES shows two local minima with respect to *j* (*ϕ* ≈ −90° and *ϕ* ≈ −13°) when the C_1_-C_2_ bond length is 1.7 Å. In comparison, PESs of substituted systems exhibit only one local minimum at *ϕ* » −90°, whereas *ϕ* ≈ 0° corresponds to the maxima for these cases. This energy penalty for the S_1_ stated at *ϕ* ≈ 0° results from steric effects, which is significantly larger for the bulky -*t*Bu group as shown in Figure 5a. For these conformations, S_1_→S_0_ transitions show LE character (π-π* transition on the anthracene moiety) for all systems regardless of *ϕ* in all cases as indicated by calculated Λ values and excited state density differences as shown in Figure 6a and 6c. As a result, excited state energetics are governed mainly by the steric hindrance of the bulky group rather than electronic effects for these conformations.

For conformations with fully elongated C_1_→C_2_ bond (2.6 Å), S_1_ PESs show quite similar trends for both substituted and unsubstituted systems (Figure 5b) unlike the PESs with unstretched C_1_→C_2_. This is related to the reduction of steric hindrance on the molecular geometries with C_1_-C_2_ bond elongation. As shown in Figure 5b, two local minima (*ϕ* ≈ −90° and *ϕ* ≈ 0°) exist in the PESs for all systems, which correspond S_1_®S_0_ transitions with HLCT and CT characters (Figures 6b and 6d) respectively. With *-*CH_3_ and *-*C_2_H_5_ substitution as well as with the unsubstituted system, parallel conformation (*ϕ* = 0°) is predicted to be energetically more favorable compared to the tilted conformation (*ϕ* = 90°), whereas tilted conformation is still energetically more favorable with -*t*Bu substitution. In addition, the rotational energy barrier is significantly larger with -*t*Bu substitution (~0.7 eV)), indicating a less probable path for the formation of dark CT state for this case.

In previous experimental and theoretical studies, it is shown that *o-*CB-H-*Ant* exhibits dual emission in solution from energetically close LE and TICT conformations on S_1_ PES [16]. However, experimentally obtained quantum yield of the florescence is quite low in solution (*Φ*PL = 0.02), which is associated with quenching through low-energy dark CT state [22,27], along with the vibrational motion of C_1_-C_2_ bond on *o-*CB. [32] Correspondingly, a single emission band with low quantum yield (*Φ*PL < 0.01) is found previously for *o-*CB-*-*CH_3_-*Ant* in solution state. In the case of bulky substituents such as -TMS (trimethylsilyl) or -*t*Bu, however, a significant increase for the quantum yield is observed for *o-*CB-*Ant* or other *o-*CB-*fluorophore* systems [12,17,38]. The increase in the emission quantum yield is often associated to the suppression of C_1_-C_2_ vibrational motion as a result of steric effects induced by bulky groups. In this investigation, it is shown that the S_1_ PESs of *o-*CB*-t*Bu*-Ant* exhibit distinct features compared to the unsubstituted or *-*CH_3_ and *-*C_2_H_5_ substituted systems. One main difference is that the excited-state energies of parallel conformations (*ϕ* ≈ 0°) with relatively short C_1_-C_2_ bonds are significantly higher for *o-*CB*-t*Bu*-Ant* compared to other systems. For these geometries, calculated E_S1_ also surpasses the initial excitation energy (E_0®1_) for *o-*CB*-t*Bu*-Ant*, indicating energetically inaccessible conformations on S_1_ PES.

Another important feature is that the twisted conformations (*ϕ* ≈ −90°) are energetically more favorable for -*t*Bu substituted system, especially for 2.2–2.6 Å C_1_-C_2_ bond range. As a result, twisted conformation is predicted to be the minima for *o-*CB*-t*Bu*-Ant* even at 2.6 Å C_1_-C_2_ bond length, meanwhile parallel conformations (dark CT state) are predicted to be the minima for the other systems with the same C_1_-C_2_ bond length. It should also be noted that the CT state of the *o-*CB*-*H*-Ant* is predicted to be the global minimum on the S_1_ PES, while CT state of *o-*CB*-*CH_3_*-Ant* and *o-*CB*-*C_2_H_5_*-Ant* are within 0.05 eV with the TICT state of the same systems. Meanwhile, the difference between TICT and CT states of *o-*CB*-t*Bu*-Ant* is 0.22 eV, where the TICT conformation is predicted to be the global minimum on the S_1_ PES. These results show that there is a substantial energy penalty for *o-*CB*-t*Bu*-Ant* to reach the parallel conformations and resulting dark CT state, which most likely constrain the molecule in the emissive twisted conformations.

## 4. Conclusion

In this contribution, TDDFT calculations were performed for the S_1_ PES of *o-*CB*-*H*-Ant*, *o-*CB*-*CH_3_*-Ant*, *o-*CB*-*C_2_H_5_*-Ant*, and *o-*CB*-t*Bu*-Ant* to understand the steric effects on the photophysical properties of *o-*CB-fluorophore systems. It is shown that a nonemissive CT state exists for all systems as a result of C_1_-C_2_ bond elongation (2.53–2.68 Å) and parallel orientation (*ϕ* ≈ 0°) between two moieties. In the case of unsubstituted system (*o-*CB*-*H*-Ant*), the adiabatic energy of this CT state is lowest on the S_1_ PES, whereas the CT state shows similar energetics with the emissive TICT state (*ϕ* ≈ −90°) for *-*CH_3_ and *-*C_2_H_5_ substituted cases. For these systems, it is seen that S_1_ PES is predicted to be quite flat for conformations especially for tilted conformations, indicating that it is possible for these systems to reach the nonemissive CT state without a large energy penalty.

In comparison, S_1_ PES of *o-*CB*-t*Bu*-Ant* clearly shows a global minimum for the conformations with *ϕ* ≈ −90° (TICT state), and the energy difference between the TICT and CT states are quite large (0.22 eV) with former being energetically favorable. In addition, adiabatic energies are significantly higher for parallel conformations (*ϕ* ≈ 0°) of *o-*CB*-t*Bu*-Ant* compared to the initial vertical excitation energy (absorption), especially for shorter C_1_-C_2_ bonds. These results show that both pathways for the nonemissive CT state are energetically unfavorable for *o-*CB*-t*Bu*-Ant* as a result of the interplay between steric and electronic effects, which results in higher emission yields even in solution state.

## Figures and Tables

**Table 1:** Comparison of 0→1 or 1→0 transition energies, oscillator strengths, energy difference between the ground state and excited state geometries (ΔE_0_), adiabatic excited state energies (ES_1_), and geometrical parameters of each conformation for o-CB-Ant derivatives.

*o-*CB-fluorophore	0→1 or 1→0 (eV)	Oscillator Strength (a.u)	ΔE_0_ (eV)	E_S1_ (eV)	C_1_-C_2_ length (Å)	*ϕ* C_1_-C_2_-C_3_-C_4_ (degrees)
Vertical transition (0®1)						
*o-*CB-H-*Ant*	3.37	0.22	0.00	3.37	1.65	−15
*o-*CB*-*CH_3_*-Ant*	3.33	0.21	0.00	3.33	1.71	−86
*o-*CB*-*C_2_H_5_*-Ant*	3.28	0.27	0.00	3.28	1.73	−87
*o-*CB*-t*Bu*-Ant*	3.28	0.22	0.00	3.28	1.80	−88
LE State (1®0)						
*o-*CB-H-*Ant*	2.61	0.25	0.36	2.97	1.66	−13
*o-*CB*-*CH_3_*-Ant*	2.55	0.24	0.39	2.94	1.71	−86
*o-*CB*-*C_2_H_5_*-Ant*	2.55	0.21	0.37	2.92	1.72	−86
*o-*CB*-t*Bu*-Ant*	2.56	0.26	0.34	2.90	1.80	−88
TICT State (1®0)						
*o-*CB-H-*Ant*	2.25	0.49	0.72	2.97	2.25	−86
*o-*CB*-*CH_3_*-Ant*	2.31	0.38	0.54	2.85	2.11	−85
*o-*CB*-*C_2_H_5_*-Ant*	2.30	0.39	0.53	2.84	2.14	−86
*o-*CB*-t*Bu*-Ant*	2.19	0.49	0.40	2.59	2.35	−86
CT State (1®0)						
*o-*CB-H-*Ant*	1.31	0.00	1.48	2.79	2.53	0
*o-*CB*-*CH_3_*-Ant*	1.23	0.00	1.63	2.87	2.59	0
*o-*CB*-*C_2_H_5_*-Ant*	1.24	0.00	1.66	2.89	2.59	0
*o-*CB*-t*Bu*-Ant*	1.13	0.00	1.68	2.81	2.68	0
